# Trimetazidine Modulates Mitochondrial Redox Status and Disrupted Glutamate Homeostasis in a Rat Model of Epilepsy

**DOI:** 10.3389/fphar.2021.735165

**Published:** 2021-10-08

**Authors:** Muhammad Y. Al-Shorbagy, Walaa Wadie, Dalia M. El-Tanbouly

**Affiliations:** ^1^ Department of Pharmacology and Toxicology, Faculty of Pharmacy, Cairo University, Cairo, Egypt; ^2^ Department of Pharmaceutical Sciences, College of Pharmacy, Gulf Medical University, Ajman, United Arab Emirates

**Keywords:** trimetazidine, mitochondrial oxidative stress, glutamate transporters, ERK1/2, astrogliosis

## Abstract

Mitochondrial oxidative status exerts an important role in modulating glia–neuron interplay during epileptogenesis. Trimetazidine (TMZ), a well-known anti-ischemic drug, has shown promising potential against a wide range of neurodegenerative disorders including epilepsy. Nevertheless, the exact mechanistic rationale behind its anti-seizure potential has not been fully elucidated yet. Herein, the impact of TMZ against mitochondrial oxidative damage as well as glutamate homeostasis disruption in the hippocampus has been investigated in rats with lithium/pilocarpine (Li/PIL) seizures. Animals received 3 mEq/kg i.p. LiCl_3_ followed by PIL (single i.p.; 150 mg/kg) 20 h later for induction of seizures with or without TMZ pretreatment (25 mg/kg; i.p.) for five consecutive days. Seizure score and seizure latency were observed. Mitochondrial redox status as well as ATP and uncoupling protein 2 was recorded. Moreover, glutamate homeostasis was unveiled. The present findings demonstrate the TMZ-attenuated Li/PIL seizure score and latency. It improved mitochondrial redox status, preserved energy production mechanisms, and decreased reactive astrocytes evidenced as decreased glial fibrillary acidic protein immune-stained areas in hippocampal tissue. In addition, it modulated phosphorylated extracellular signal-regulated kinases (*p*-ERK1/2) and p-AMP–activated protein kinase (*p*-AMPK) signaling pathways to reflect a verified anti-apoptotic effect. Consequently, it upregulated mRNA expression of astroglial glutamate transporters and reduced the elevated glutamate level. The current study demonstrates that TMZ exhibits robust anti-seizure and neuroprotective potentials. These effects are associated with its ability to modulate mitochondrial redox status, boost *p*-ERK1/2 and *p*-AMPK signaling pathways, and restore glutamate homeostasis in hippocampus.

## Introduction

Mitochondrial oxidative stress is the leading cause of age-related degenerative diseases and may contribute profoundly to seizure instigation (Beal, 2005; Waldbaum and Patel, 2010). Oxidative mitochondrial damage accelerates neuronal excitability *via* affecting mitochondrial roles crucial for normal brain functioning (Rowley and Patel, 2013). The deleterious role of mitochondrial dysfunction in epileptogenesis stems from the correlation between epilepsy and the frequent incidence of inherited mitochondrial disorders such as those occurring with childhood encephalopathies (Waldbaum and Patel, 2010). Parallel to the impediment of sufficient adenosine triphosphate (ATP) production by neurons, the resultant mitochondrial damage affects antioxidant defenses, fatty acid oxidation, neurotransmitter biosynthesis, regulation of cytosolic Ca^2+^, and synaptic glutamate homeostasis (Waldbaum and Patel, 2010). Mitochondrial dysfunction is, hence, implicated in excessive neuronal excitability and epileptogenesis. It accelerates apoptotic neuronal death following glutamate excitotoxicity, which independently participates in seizure-induced neuronal loss especially in the hippocampus (Nicholls and Ward, 2000; Noh et al., 2006). The inner mitochondrial membrane protein, uncoupling protein 2 (UCP2), could largely preserve mitochondrial membrane potential and hamper mitochondrial oxidative stress through controlling mitochondrial-derived reactive oxygen species (Pierelli et al., 2017).

Glutamate transporters are responsible for preserving synaptic glutamate in non-neurotoxic levels (Todd and Hardingham, 2020). Though they are highly expressed on both neurons and neuroglia, it has been supposed that astroglial transporters, namely, GLT-1 and GLAST, are responsible for the uptake of the majority of glutamate (Danbolt, 2001). Their dysfunction may accordingly contribute to an increase in glutamate beyond the normal levels, promoting seizure development. This was further verified when the use of threo-β-benzyloxyaspartate, a glutamate transport inhibitor, extended epileptiform activity (Shimamoto et al., 1998). GLT-1 knockout mice develop spontaneous seizures (Tanaka et al., 1997), while those lacking GLAST show high susceptibility to seizures (Ueda et al., 2002). GLT-1 and GLAST hypofunction or hypo-expression has been observed with aging and is indeed implicated in several neurodegenerative disorders, including epilepsy (Todd and Hardingham, 2020). The age-related decline in astroglial glutamate transporters in mitochondrial superoxide dismutase knockout mice coincided with epileptic seizure susceptibility (Liang and Patel, 2004). Glutamate transporters are highly vulnerable to oxidative damage resulting in the impairment of glutamate uptake machinery (Trotti et al., 1998). These observations imply that mitochondrial oxidative stress and secondary dysfunction may be sufficient to increase seizure vulnerability through modifications of astroglial glutamate transporters.

Trimetazidine [1-(2,3,4-trimethoxybenzyl) piperazine dihydrochloride] (TMZ) is mostly used as an anti-ischemic drug. It optimizes oxygen demands *via* shifting fatty acid oxidation to glucose oxidation (Peng et al., 2014). Potentiation of glucose oxidation in an ischemic cell preserves cellular ATP, as energy obtained during glucose oxidation requires less oxygen than fatty acid oxidation (Sandhiya, 2015). TMZ was reported to prevent cerebral ischemia-reperfusion injury in experimental animals (Dhote and Balaraman, 2008). It improved brain activity by increasing brain glucose uptake (Nowak et al., 2006), conserving brain mitochondrial membrane, and protecting neuronal cells against intracellular acidosis (Jain et al., 2010). It also augmented the central ATP level (Al-Kuraishy and Al-Gareeb, 2017) and hindered hippocampal oxidative damage *via* the upregulation of antioxidant enzymes and inhibition of lipid peroxidation in an animal model of Alzheimer’s disease (Hassanzadeh et al., 2015). TMZ profoundly elevated seizure threshold in the increasing-current electroshock seizure test (Jain et al., 2010) and prevented pentylenetetrazol (PTZ)-induced kindling in mice (Jain et al., 2011). The exact mechanistic rationale behind its anti-seizure potential, however, has not been fully elucidated yet.

The current study was, therefore, conducted to investigate whether TMZ could modulate mitochondrial redox status and glutamate homeostasis in the hippocampi of rats following lithium/pilocarpine (Li/PIL) injection. It also explored the impact of such effects on the incidence and intensity of induced seizures.

## Materials and Methods

### Animals

Adult male Wistar rats each weighing 180 ± 200 g were obtained from the National Research Centre in Cairo and housed at constant temperature and humidity at the animal facility of the Faculty of Pharmacy, Cairo University. Seizure induction was done from 9 am to 12 pm to diminish circadian effects on seizure vulnerability. The investigation fulfilled the Guide for the Care and Use of Laboratory Animals of the Ethical Committee for Animal Experimentation at Faculty of Pharmacy, Cairo University (Permit number: PT2715).

### Drugs and Chemicals

Lithium chloride (LiCl_3_) and pilocarpine (PIL) were purchased from Sigma-Aldrich, MO, United States, while trimetazidine dihydrochloride (TMZ) was purchased from Rameda Pharmaceutical Company, Cairo, Egypt.

### Experimental Design

Animals were randomly allocated into four groups each of 15 rats. Group I received saline i.p. to serve as the normal control group. Group II (normal + TMZ) received TMZ (25 mg/kg; i.p.) for five consecutive days. Group III (Li/PIL control) received LiCl_3_ in a dose of 3 mEq/kg i.p. followed by PIL (single i.p.; 150 mg/kg) 20 h later, for the induction of seizures (Al-Shorbagy et al., 2013; El-Sayed et al., 2021). Group IV (Li/PIL + TMZ) was pretreated with TMZ (25 mg/kg; i.p.) for five consecutive days before receiving Li/PIL at the same regimen as Group III.

Immediately after PIL injection, rats were placed individually in Plexiglas cages and were observed for 30 min. Each rat was assigned a convulsive score from 0 to 5 based on the Racine scale (Racine, 1972), where behavioral arrest, hair-raising excitement, and rapid breathing are denoted by 0; mouth movements (lips and tongue), vibrissae movements, and salivation are denoted by 1; head and eye clonus is denoted by 2; forelimb clonus and “wet dog shakes” are denoted by 3; clonic rearing is denoted by 4; clonic rearing with a loss of postural control and uncontrollable jumping is denoted by 5. Stage 3–5 seizure latency and median seizure stage as well as seizure incidence were recorded. Animals that did not show Stage 3–5 seizure within the observation period were given a maximum latency of 30 min (Al-Shorbagy et al., 2013; El-Sayed et al., 2021). No mortality was recorded during the observation period.

Four hours after PIL injection, rats were euthanized by decapitation under light anesthesia, and their brains were carefully excised. Brains of three rats from each group were preserved in 10% formalin to be used for histological examinations and immunohistochemical staining. The hippocampi of the remaining rats (*n* = 12/group) were isolated and divided into two subsets. The hippocampi in the first subset (n = 6/group) were homogenized in cold phosphate buffer saline (PBS; pH = 7.4). In the second subset (*n* = 6/group), the hippocampi were frozen at −80°C to be used in Western blot and quantitative real-time PCR (qRT-PCR) analyses.

### Isolation of Mitochondria-Rich Fraction

The homogenized hippocampal tissues were centrifuged with 0.25 M sucrose at 2,000 × *g* for 10 min at 4°C. Pellets were discarded, and 0.75 M sucrose in (4-(2-hydroxyethyl)-1-piperazineethanesulfonic acid) HEPES buffer was added to the supernatant and centrifuged at 10,000 × *g* for 30 min. HEPES buffer was added to the mitochondria pellets after discarding the supernatant and recentrifuged for 10 min at 10,000 × *g*, then the supernatant was discarded and PBS was added to the final mitochondria-rich fraction pellets (Ahmed and El-Maraghy, 2013; Ahmed et al., 2014). This was stored at −80°C to be used for determination of UCP2 and as oxidative stress biomarkers.

### Biochemical Measurements

#### Mitochondrial Oxidative Stress Biomarkers

The oxidative stress status was estimated in the mitochondria-rich fraction by measuring malondialdehyde (MDA), an end product of lipid peroxidation, using the specific colorimetric kit (Biodiagnostics, Egypt) and expressed as nmol/mg protein. Reduced glutathione (GSH) was measured according to the method of Ellman (1959) and expressed as nmol/mg protein. In addition, the total antioxidant capacity (TAC) was determined colorimetrically, as it considers the cumulative effect of all the antioxidants present in the hippocampal mitochondrial fraction (Biodiagnostics, Egypt) and is expressed as mmol/mg protein.

#### Adenosine Triphosphate Content

The ATP content was estimated in the total hippocampal homogenate using the BioVision's ATP colorimetric assay kit (BioVision, Milpitas, United States). The assay utilizes the phosphorylation of glycerol to generate a product that is easily quantified colorimetrically at 570 nm and expressed as pg/mg protein.

#### Enzyme Linked Immunosorbent Assay

The total tissue homogenate was used to determine glutamate and cytochrome c (Cyt c) levels using ELISA assay kits (MyBioSource, San Diego, United States, and Elabscience, Texas, United States, respectively). Their results were expressed as µg/mg protein and pg/mg protein, respectively. In addition, UCP2 was measured in the mitochondrial rich fraction by a specific ELISA kit (Cusabio, Texas, United States) and expressed as pg/mg protein.

#### Caspase-3 Activity

Caspase-3 activity was estimated in the total hippocampal homogenate using the caspase-3 colorimetric assay kit (Elabscience, Texas, United States). The assay depends on the dissociation of yellow group *p*-nitroaniline (pNA) from caspase-3 sequence-specific peptides. pNA has an absorption peak which is easily measured colorimetrically at 405 nm and used to express caspase-3 activity as nmol pNA/h/mg protein.

#### Western Blotting Analysis

Following total hippocampal protein quantification by using the Bradford kit (Bio-Rad Protein Assay Kit, CA, United States), 20 μg protein of each sample was separated by SDS/polyacrylamide gel electrophoresis, then transferred onto polyvinylidene difluoride membranes (Thermo Fischer Scientific, MA, United States). A blocking solution comprising 20 mM Tris-Cl, pH 7.5, 150 mM NaCl, 0.1% Tween 20, and 3% bovine serum albumin (BSA) was then added to the membranes at room temperature for 2 h to avoid nonspecific binding of the antibodies before incubating them overnight at 4°C with one of the following primary antibodies: p-extracellular signal-regulated kinases (p-44/42 ERK1/2) (Thr202/Tyr204), p-AMP-activated protein kinase (*p*-AMPK) (Thy 172), or Beta actin (β-actin) primary antibodies (Thermo Fisher Scientific, MA, United States). Next, the membranes were probed with horseradish peroxidase (HRP)-conjugated secondary antibodies (Thermo Fisher Scientific, MA, United States). Finally, the bands were established with the Chemiluminescence Reagent Kit (Amersham Biosciences, IL, United States), and their intensity was analyzed by Chemi Doc™ Imaging System with Image Lab™ software version 5.1 (Bio-Rad Laboratories Inc., Hercules, CA, United States). All values were normalized to that of β-actin and presented as fold-change.

#### Quantitative Real-Time PCR Analysis of Glial Glutamate Transporters

An extraction kit (Qiagen, Germantown, MD, United States) was used to extract total RNA from the total hippocampal tissue, which was then measured spectrophotometrically at 260 nm. Equal amounts of the extracted RNA were then reverse transcribed into cDNA using a high-capacity cDNA reverse transcription kit (Thermo Fischer Scientific, MA, United States). To assess the gene expression of glial glutamate transporters, GLAST and GLT-1, qRT-PCR was performed using ABI PRISM 7500 Fast Sequence Detection System (Applied Biosystems, CA, United States). The sequences of the PCR primer pairs used were the following: For GLAST, F:5’- CCA​GTG​CTG​GAA​CTT​TGC​CT -3′, R: 5′- TAAAGGG CTGTACCATCCAT -3′, for GLT-1 F:5’-ACAAAAAGCAACGG AGAAGAGCC -3′, R: 5′- TAC​GGT​CGG​AGG​GCA​AAT​CC -3′, and β-actin (F: 5-TAT​CCT​GGC​CTC​ACT​GTC​CA-3′, R:5′-A ACG​CAG​CTC​AGT​AAC​AGT​C-3′). In brief, 1 μg of the total RNA was mixed with 50 μM oligo (dT) 20, 50 ng/μL random primers, and 10 mM dNTP mix in a total volume of 10 μL in an optical 96-well plate using universal cycling conditions (5 min at 95°C followed by 45 cycles of 5 s at 95°C and 10 s at 60°C). The relative expression of the target genes was obtained using the 2−ΔΔCT formula (Pfaffl, 2001). All values were normalized to that of β-actin and presented as fold-change (Livak and Schmittgen, 2001).

#### Histopathological Analysis

Brain samples fixed in 10% neutral buffered formalin were trimmed and processed in serial grades of ethanol, cleared in xylene, infiltrated with synthetic wax, and embedded out into Paraplast tissue-embedding media. 3- to 5-μ-thick sagittal sections were cut by a rotatory microtome. The sections were stained with Harris Hematoxylin and Eosin (H&E) as a general tissue examination staining method. Hippocampal neurons were outlined, then the pathological changes in the different hippocampal regions were examined at high power (×400 magnification) in each group. The Lesion scoring system for pathological changes in different hippocampal regions was performed (Al-Sayed et al., 2020). Each animal was given a Lesion score between 0 and 3, for each of the three parameters, viz., neuronal damage, perineuronal edema, and glial cells infiltrates, where, 0 indicates no change, 1 indicates mild change (less than 15% of the examined samples), 2 indicates moderate change (16–35% of examined samples), and 3 indicates severe change (more than 35% of examined samples). Total histology lesion scores, the maximum being 9, were obtained by summing the scores of the three parameters for each animal. In addition, toluidine blue stain was used for the demonstration of intact neurons count with intact subcellular and nuclear details in the different hippocampal zones.

#### Immunohistochemical Assay of Glial Fibrillary Acidic Protein

Deparaffinized 5-μ-thick tissue sections were treated with 3% hydrogen peroxide for 20 min, washed by PBS, then incubated with anti–glial fibrillary acidic protein (GFAP) monoclonal antibody from Thermo Fischer Scientific, MA, United States (1:100) overnight. After that, tissue sections were washed by PBS and incubated with a secondary antibody HRP EnVision kit (DAKO) for 20 min and then with diaminobenzidine for 10 min. Finally, they were counter stained with hematoxylin, dehydrated, and cleared in xylene for microscopic analysis.

#### Microscopic Analysis

Six random nonoverlapping fields from different hippocampal regions per tissue sample were analyzed for determination of average area % of expression of GFAP in immunostained sections, as well as mean intact neurons count in Cornu Ammonis regions (CA1, CA3) and Dentate Gyrus (DG) hilar cells. All micrographs and data were obtained by using a full HD microscope camera operated by the Leica application module for tissue sections analysis (Leica Microsystems GmbH, Wetzlar, Germany)”.

### Statistical Analyses

All data obtained, except for total histology scores, Racine scores, and seizure incidence, were expressed as mean ± standard deviation (SD). Results were analyzed using the two-way analysis of variance test (ANOVA) followed by the Tukey–Kramer multiple comparison’s test as a post-hoc test. Total histology scores and Racine scores were presented as median and analyzed using the Kruskal–Wallis test followed by Dunn's test as a post-hoc test. Incidence of Stage 3–5 seizures was compared using Fisher's exact probability test for all statistical tests, the level of significance was set at *p* < 0.05. GraphPad Prism^®^ software package, version 5 (GraphPad Software, Inc., United States), was used to carry out all statistical tests.

## Results

### Racine Score and Seizure Latency

**FIGURE 1:**
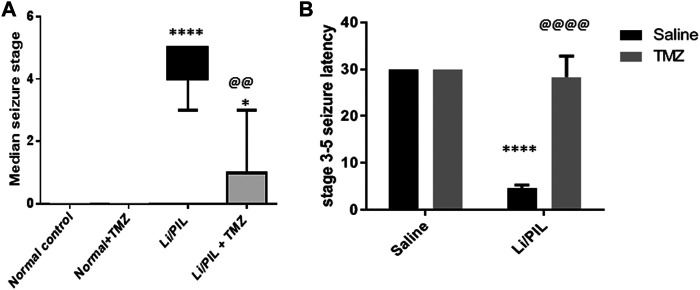
Effect of TMZ on Li/PIL-induced seizures. **(A)** Racine score, data are expressed as box plots of the median of 15 animals. Statistical analysis was done using the Kruskal–Wallis test followed by Dunn's test. **(B)** Seizure latency, data are expressed as mean of 15 animals ± SD. Two-way ANOVA followed by the Tukey–Kramer post-hoc test was used for statistical analyses. *Significantly different from the normal control group (Saline) at *p* < 0.05; ****Significantly different from the normal control group (Saline) at *p* < 0.0001; ^@@^Significantly different from the Li/PIL control group at *p* < 0.01; ^@@@@^Significantly different from the Li/PIL control group at *p* < 0.0001.

As shown in Figures 1A,B, Stage 3–5 seizures were reached in all rats subjected to Li/PIL, within nearly 5 min after seizure induction. However, TMZ succeeded in delaying seizure latency to about 28.3 min (*p* < 0.0001) and noticeably reduced seizure incidence (*p* < 0.0001) and severity (*p* < 0.01) by 86.6 and 78.6%, respectively.

### Effect of Trimetazidine on Hippocampal mRNA Expression of GLT-1 and GLAST

**FIGURE 2:**
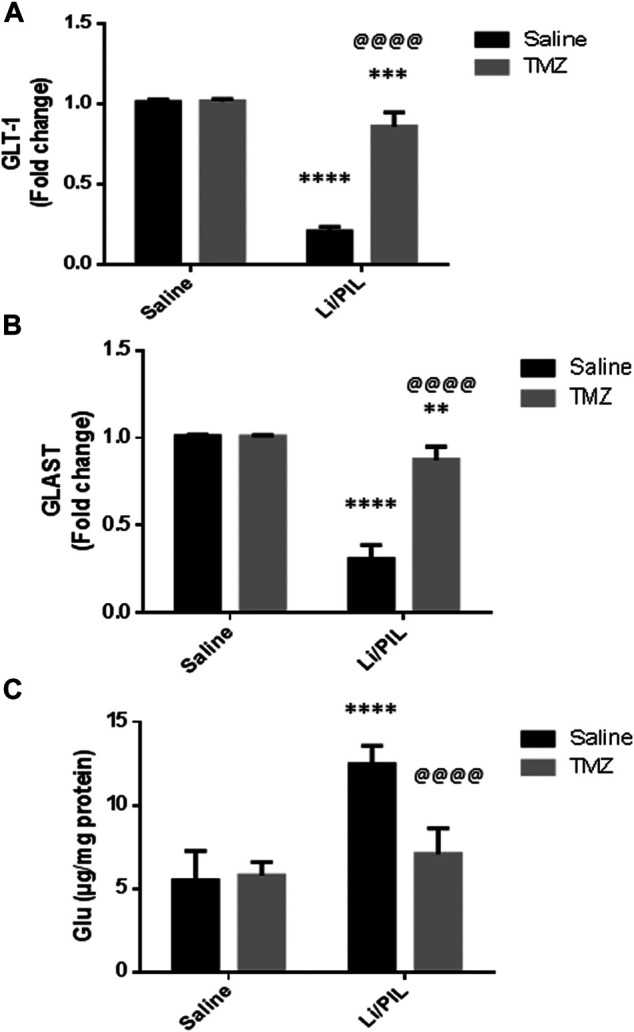
Effect of TMZ on glial glutamate transporters and hippocampal glutamate level in rats with Li/PIL-induced seizures. **(A)** hippocampal mRNA expression of GLT-1 as indicated by qRT-PCR analysis, **(B)** hippocampal mRNA expression of GLAST as indicated by qRT-PCR analysis, **(C)** hippocampal glutamate level. Values are expressed as mean of six animals ± SD. Two-way ANOVA followed by the Tukey–Kramer post-hoc test was used for statistical analyses of data. **Significantly different from the normal control group (Saline) at *p* < 0.01; ***Significantly different from the normal control group (Saline) at *p* < 0.001; ****Significantly different from the normal control group (Saline) at *p* < 0.0001; ^@@@@^Significantly different from the Li/PIL control group at *p* < 0.0001.

Hippocampal mRNA expression of GLT-1 and GLAST was prominently reduced reaching 20 and 30%, respectively, as compared to the normal control (*p* < 0.0001). Pretreatment with TZM, however, markedly increased their mRNA expression to reach 4-fold and 2.8-fold to that in the Li/PIL control group, respectively (*p* < 0.0001) (Figures 2A,B).

### Effect of Trimetazidine on Hippocampal Glutamate Level

As shown in Figure 2C, Li/PIL induced an increase in the hippocampal glutamate level, reaching 2.2-fold to that in the normal control group (*p* < 0.0001 On the contrary, rats pretreated with TMZ exhibited a normal level of glutamate in their hippocampi, *p* = 0.21, recording a decrease of 43.2%, as compared to the Li/PIL control group.

### Effect of Trimetazidine on Hippocampal GFAP Expression

**FIGURE 3:**
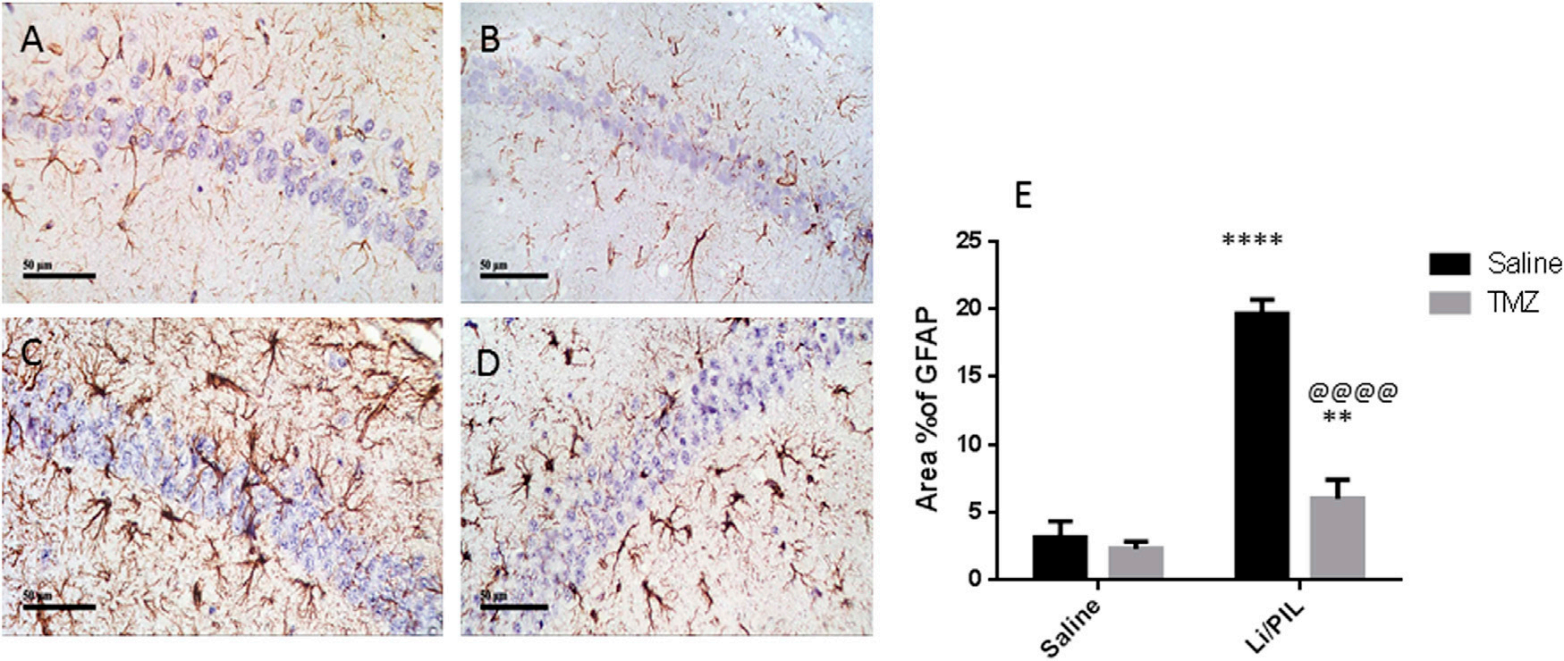
Effect of TMZ on GFAP expression in the hippocampi of rats with Li/PIL-induced seizures as indicated by immunohistochemistry (×400 original magnification). Normal control rats **(A)**, Normal + TMZ rats **(B)**, Li/PIL control rats **(C)**, and TMZ + Li/PIL rats **(D)**. Area % of GFAP **(E)**. Values are expressed as mean of three animals ± SD. Two-way ANOVA followed by the Tukey–Kramer post-hoc test was used for statistical analyses of data. **Significantly different from the normal control group (Saline) at *p* < 0.01; ****Significantly different from the normal control group (Saline) at *p* < 0.0001; ^@@@@^Significantly different from the Li/PIL control group at *p* < 0.0001.

Li/PIL induced astrocytes activation as demonstrated by the obvious increase in the immunostaining density of hippocampal GFAP (19.67 ± 1.03 vs 3.2 ± 1.1, respectively), an effect that was largely prevented by TMZ recording a decrease of 69.5%, as compared to the Li/PIL control group (Figure 3).

### Effect of Trimetazidine on Hippocampal p-44/42 ERK1/2 (Thr202/Tyr204) and *p*-AMPK (Thy172)

**FIGURE 4:**
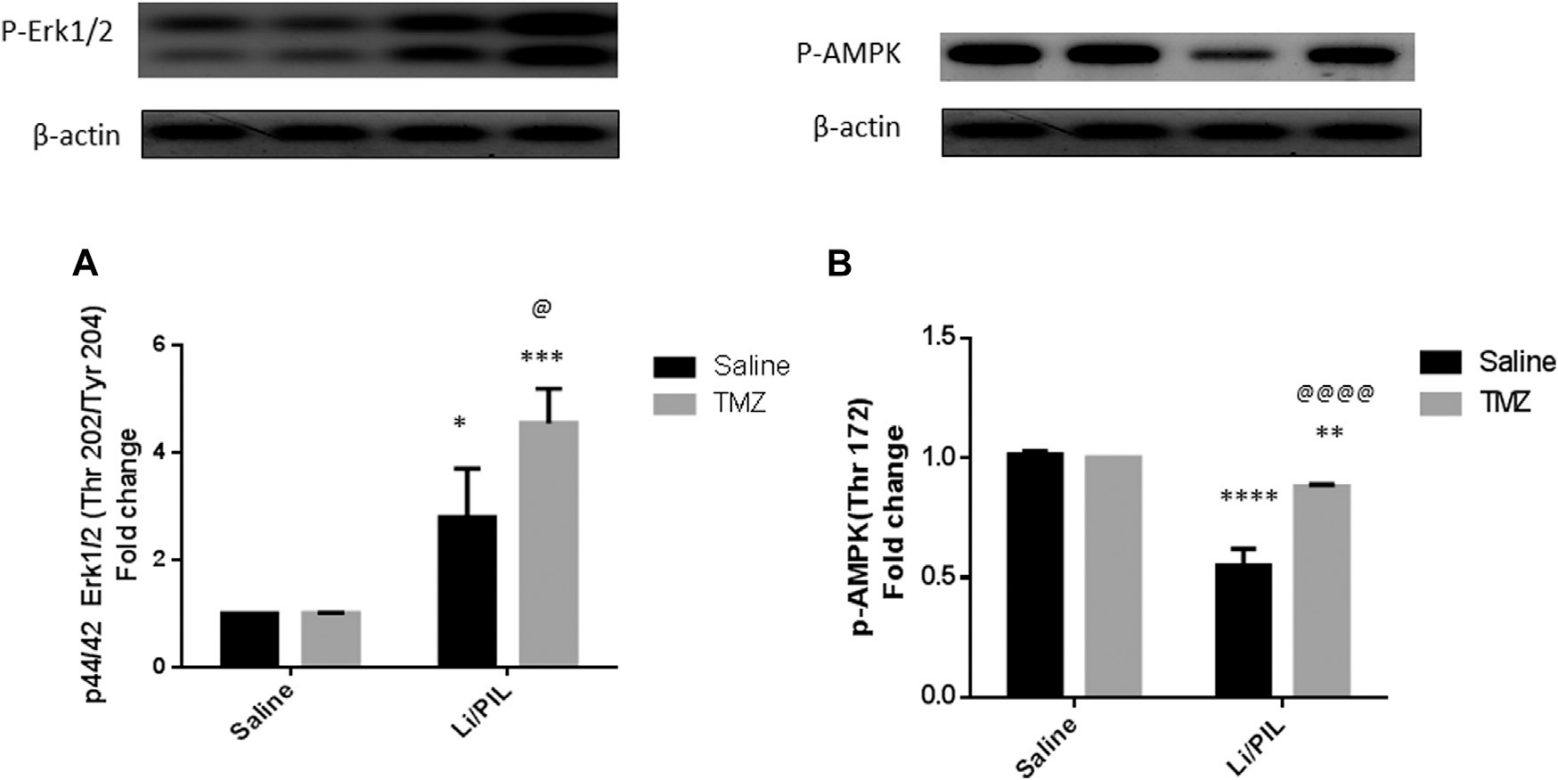
Effect of TMZ on the protein expression of p-44/42 Erk1/2 (Thr202/Tyr204) and *p*-AMPK (Thy172) in the hippocampi of rats with Li/PIL-induced seizures. **(A)** The expression of p-44/42 Erk1/2 (Thr202/Tyr204) as indicated by Western blot analysis; **(B)** the expression of *p*-AMPK (Thy172) as indicated by Western blot analysis. Values are expressed as mean of three animals ± SD. Two-way ANOVA followed by the Tukey–Kramer post-hoc test was used for statistical analyses of data. *Significantly different from the normal control group (Saline) at *p* < 0.05; **Significantly different from the normal control group (Saline) at *p* < 0.01; ***Significantly different from the normal control group (Saline) at *p* < 0.001; ****Significantly different from the normal control group (Saline) at *p* < 0.0001; ^@^Significantly different from the Li/PIL control group at *p* < 0.05; ^@@@@^Significantly different from the Li/PIL control group at *p* < 0.0001.

Rats subjected to Li/PIL exhibited a marked rise in hippocampal p-44/42 ERK1/2 (2.8 ± 0.9 vs 1 ± 0.0) along with a profound decline in *p*-AMPK (0.55 ± 0.07 vs 1.015 ± 0.01) as compared to that in the normal control (*p* < 0.0001). TMZ induced a further increase in hippocampal *p*-ERK1/2 by 62.5% and prevented the drop in *p*-AMPK reaching 1.6-fold to that in the Li/PIL control group (*p* < 0.05 and *p* < 0.0001, respectively) (Figures 4A,B).

### Mitochondrial Oxidative Stress in Hippocampus

**FIGURE 5:**
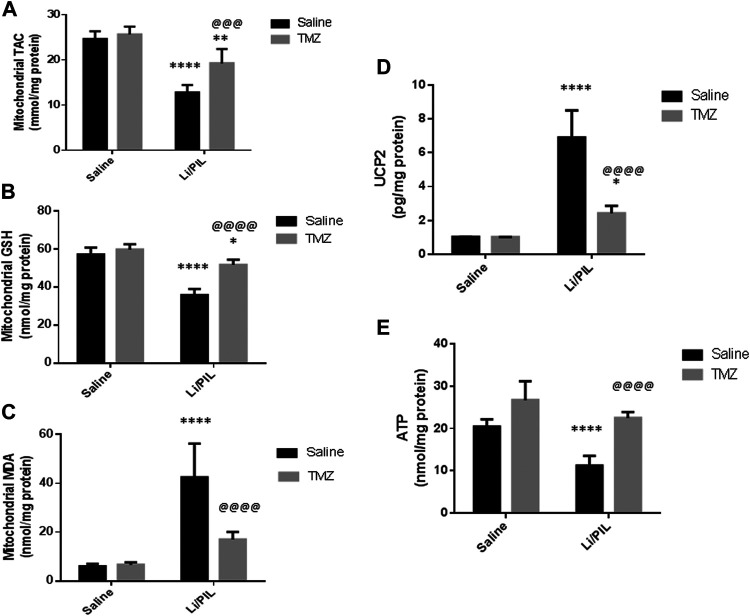
Effect of TMZ on mitochondrial oxidative stress, mitochondrial uncoupling protein 2 (UCP2), and ATP production in the hippocampi of rats with Li/PIL-induced seizures. **(A)** TAC, **(B)** reduced GSH contents, **(C)** MDA levels, **(D)** UCP2, **(E)** ATP. Values are expressed as mean of six animals ± SD. Two-way ANOVA followed by the Tukey–Kramer post-hoc test was used for statistical analyses of data. *Significantly different from the normal control group (Saline) at *p* < 0.05; **Significantly different from the normal control group (Saline) at *p* < 0.01; ****Significantly different from the normal control group at *p* < 0.0001; ^@@@^Significantly different from the Li/PIL control group at *p* < 0.001; ^@@@@^Significantly different from the Li/PIL control group at *p* < 0.0001.

Induction of seizures was accompanied by a profound disruption in mitochondrial oxidative homeostasis in the hippocampi as manifested by the depletion of the mitochondrial TAC (12.8 ± 1.6 vs 24.7 ± 1.6) and GSH (35.9 ± 1.2 vs 57.1 ± 3.6) content along with the marked increase in mitochondrial MDA (42.5 ± 13.7 vs 6.1 ± 0.97 as compared to that in the normal control group (*p* < 0.0001). Pretreatment with TMZ effectively enhanced the mitochondrial TAC as well as GSH content to reach 1.5-fold and 1.44-fold, respectively, to that in the Li/PIL group (*p* < 0.001 and *p* < 0.0001). Consequently, TMZ reduced mitochondrial MDA production by 60% as compared to Li/PIL rats (*p* < 0.0001), showing a similar level to that in the normal control group *p* = 0.065 (Figures 5A–C).

### Effect of Trimetazidine on Mitochondrial Uncoupling Protein 2 and ATP Production

Li/PIL increased UCP2 in mitochondria-rich fraction (6.9 ± 1.6 vs 1.02 ± 0.02) with a pronounced reduction in the hippocampal ATP (11.2 ± 2.3 vs 20.5 ± 1.7) content (*p* < 0.0001). However, TMZ was effective in impeding UCP2 expression in mitochondria-rich fraction by 65% as compared with the Li/PIL control group (*p* < 0.0001) and restoring hippocampal ATP, reaching a comparable content to the normal control group, *p* = 0.59, to reach 1.9-fold to that in the Li/PIL control group (Figures 5D,E).

### Effect of Trimetazidine on Hippocampal Cyt c and Caspase-3 Activity

**FIGURE 6:**
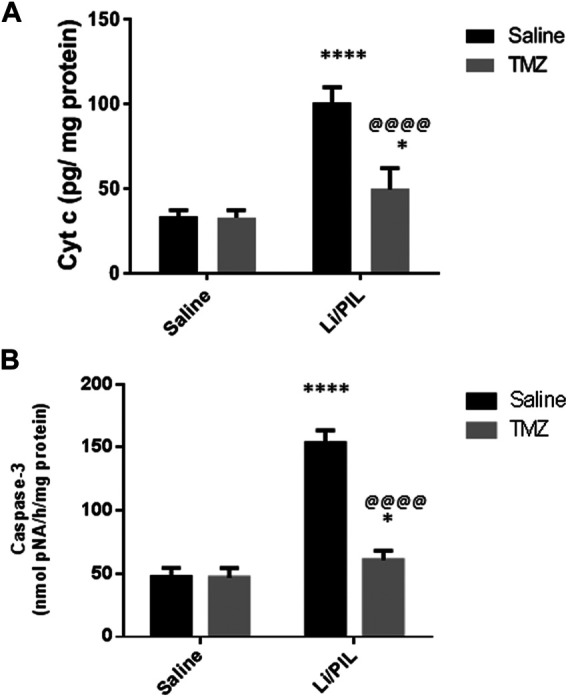
Effect of TMZ on the Cyt c content and caspase-3 activity in rats with Li/PIL-induced seizures. **(A)** Cyt c content, **(B)** caspase-3 activity. Values are expressed as mean of six animals ± SD. Two-way ANOVA followed by the Tukey–Kramer post-hoc test was used for statistical analyses of data. *Significantly different from the normal control group (Saline) at *p* < 0.05; ****Significantly different from the normal control group (Saline) at *p* < 0.0001; ^@@@@^Significantly different from the Li/PIL control group at *p* < 0.0001.

Injection of Li/PIL was associated with a massive disruption in the mitochondrial inner membrane as revealed by the marked elevation in the hippocampal Cyt c content (100.9 ± 8.9 vs 33.4 ± 3.8) and, sequentially, in caspase-3 activity (153.6 ± 9.8 vs 47.5 ± 7.1), as a marker of apoptotic cell death as compared to the normal control group (*p* < 0.0001). Pretreatment with TMZ largely prevented these mitochondrial derangements recording a decrease by 50.8 and 60.4%, respectively, as compared to that in the Li/PIL control group (Figures 6A,B).

### Effect of Trimetazidine on Histopathological Alterations and Hippocampal Neuronal Integrity

**FIGURE 7:**
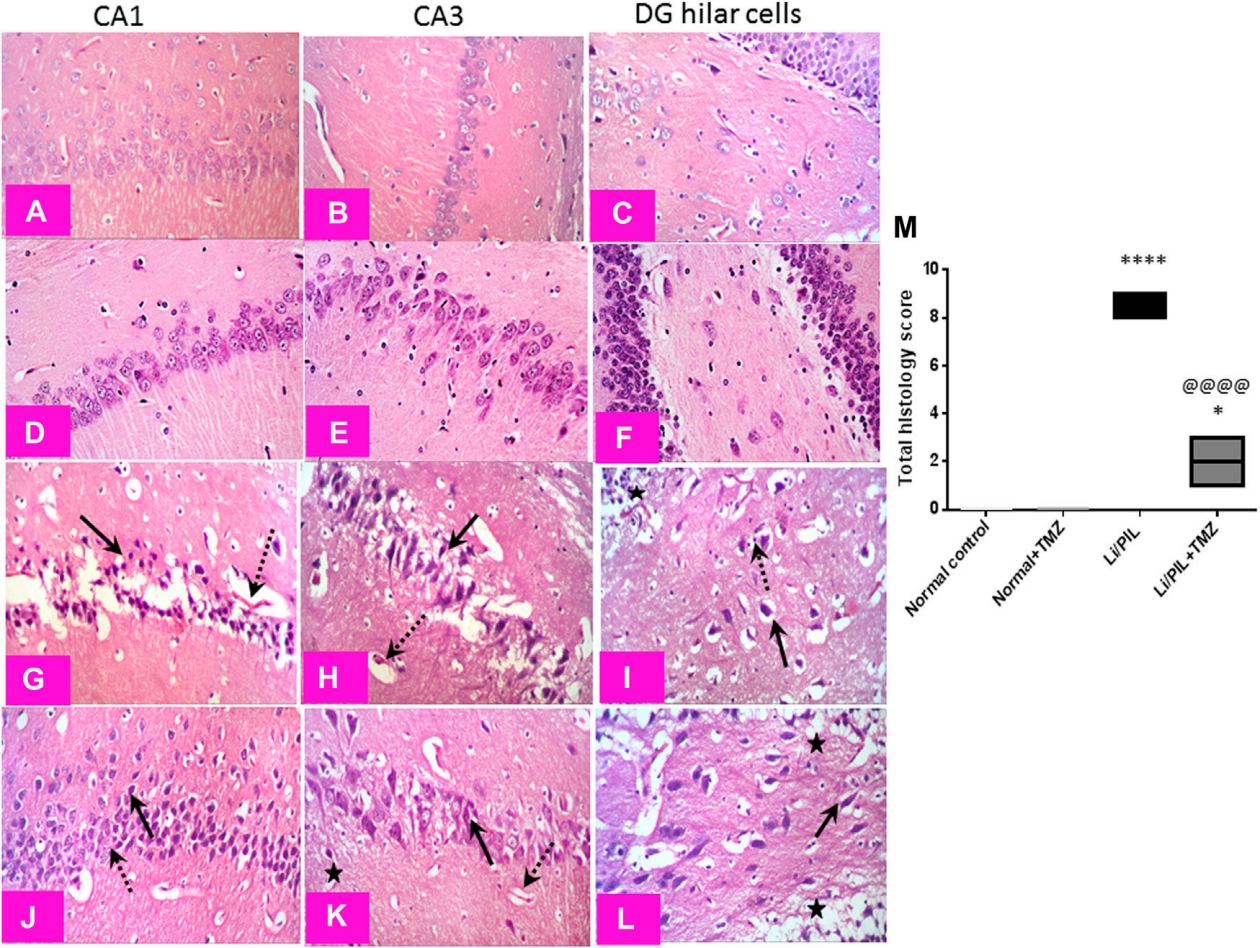
Effect of TMZ on histopathological alterations in the hippocampi of rats with Li/PIL-induced seizures (×400 original magnification). Normal control rats and normal rats treated with TMZ showed normal histology of CA1, CA3, and hilar regions **(A–C)** and **(D–F)**, respectively. Li/PIL control showed a significant loss of pyramidal cells layer (dotted arrows), many apoptotic figures, edema (star), and sloughing of capillary endothelium in CA1 and CA3 regions (arrows); in addition to many degenerated neurons (arrow), pyknotic nuclei, and glial cells infiltration (dotted arrow) **(G,H)**, and edematous fluid effusion and congested blood capillaries in the hilar region **(I)**. TMZ + Li/PIL rats demonstrated partial protection with less edema fluid and congested capillaries (star), more organizing effect on pyramidal cells in CA1 and CA3 regions with mixed arrangement of fewer damaged (arrow) and intact (dotted arrow) neurons **(J,K)**, and lower number of degenerated and apoptotic neurons in the hilar region (arrow) **(L)**. **(M)** Total histology lesion score; data are expressed as box plots of the median of three animals. Statistical analysis was done using the Kruskal–Wallis test followed by Dunn's test. *Significantly different from the normal control group (Saline) at *p* < 0.05; ****Significantly different from the normal control group (Saline) at *p* < 0.0001; ^@@@@^Significantly different from the Li/PIL control group at *p* < 0.0001.

**FIGURE 8:**
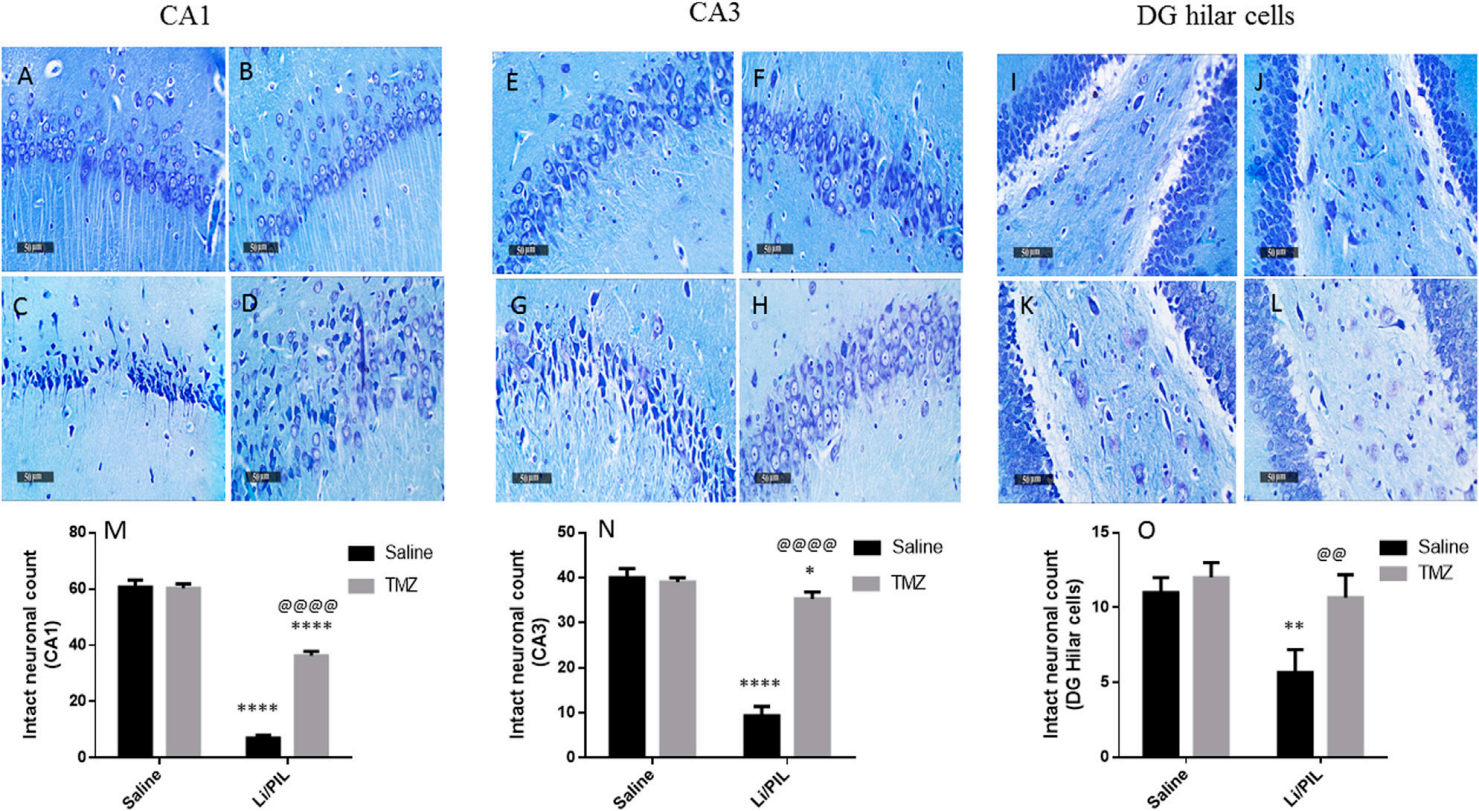
Effect of TMZ on hippocampal neuronal loss of rats with Li/PIL-induced seizures. Photomicrographs demonstrating toluidine blue–stained hippocampal tissue sections of CA1 and CA3 sub regions and DC hilar cells in normal control rats **(A,E,I)**, TMZ control rats **(B,F,J)**, Li/PIL control rats **(C,G,K)**, and TMZ + Li/PIL rats **(D,H,L)**. Intact neurons count in CA1 **(M)**, CA3 **(N)**, and DG hilar cells **(O)**. Values are expressed as mean of three animals ± SD. Two-way ANOVA followed by the Tukey–Kramer post-hoc test was used for statistical analyses of data. *Significantly different from the normal control group (Saline) at *p* < 0.05; **Significantly different from the normal control group (Saline) at *p* < 0.01; ****Significantly different from the normal control group (Saline) at *p* < 0.0001; ^@@^Significantly different from the Li/PIL control group at *p* < 0.01; ^@@@@^Significantly different from the Li/PIL control group at *p* < 0.0001.

Microscopical examination of the hippocampi from normal control rats showed the normal architecture of the CA regions (Figures 7A,B) and DG region (Figure 7C). The mean intact pyramidal neurons count was almost 61 cells/field, 40 cells/field, and 11 cells/field in CA1, CA3, and DG hilar cells, respectively, in toluidine blue-stained tissue sections (Figures 8,E,I). Rats subjected to Li/PIL exhibited a significant loss of pyramidal cells layer in CA1 and CA3 regions with many apoptotic figures associated with edema and sloughing of capillary endothelium (Figures 7G,H). Examination of the hilar region of DG revealed many degenerated neurons with pyknotic nuclei, glial cells infiltration with edematous fluid effusion, and congested blood capillaries (Figure 7I). The mean intact neurons count was almost 7 cells/field, 9 cells/field, and 6 cells/field in CA1, CA3, and DG hilar cells, respectively (Figure 8C,G,K). Pretreatment with TMZ demonstrated partial protection with more organizing effect on the pyramidal cells region of CA1 and CA3. Edema fluid and congested capillaries were less evident in all layers than in the Li/PIL group (Figure 7J,K). The hilar region examination showed a lower number of degenerated and apoptotic neurons (Figure 7L). The mean intact neurons count was almost 36 cells/field, 35 cells/field, and 11 cells/field in CA1, CA3, and DG hilar cells, respectively (Figure 8D,H,L).

## Discussion

The current investigation emphasizes the efficacy of TMZ in reducing the incidence and severity of seizures in rats subjected to Li/PIL. This anti-seizure potential was associated with a marked reduction in the hippocampal glutamate content along with a noticeable upregulation of the gene expression of astroglial transporters: GLT-1 and GLAST. These effects were correlated with the ability to preserve the integrity of neurons in the different hippocampal regions.

Disrupting the complex neuroglial circuitry has been shown to robustly contribute to neurological disorders including epilepsy (Diaz Verdugo et al., 2019). Any change in the efficiency of glutamate clearance, especially by astrocytic phenotypes, largely affects synaptic function and accordingly seizures susceptibility (Wallraff et al., 2006). The depletion of astrocytic transporters in various neurodegenerative diseases supports the strong association between the accumulation of neurotoxic levels of glutamate and reactive astrogliosis (Rothstein et al., 2005). Striking morphological and function changes are detected in reactive astrocytes altering neurotransmitter homeostasis to provoke neuronal hyperexcitability (Seifert and Steinhäuser, 2013). Preictal astrocyte activation that shortly begins after a brain insult is likely to be an adaptive response to remove damaged tissues and restore normal function (Sanz and Garcia-Gimeno, 2020). Consistently, initial astrocytes activity is directed to hamper neural activity outbreaks (Diaz Verdugo et al., 2019). During this state, the highly synchronous glial cells activity ensures their homeostatic function. Glial cells networks can efficiently reallocate (Danbolt, 2001) and absorb excessive cations and glutamate by various astrocytes transporters (Pines et al., 1992). Nevertheless, excessive astrogliosis exhausts this homeostatic function and exacerbates inflammatory response, triggering hyperexcitability of neurons and generalized seizures. The mounting efflux of K^+^ around astrocytes accompanied with glutamate uptake reverses the working way of transporters and abruptly alters the harmonized glia–neuron interaction (Danbolt, 2001).

In the current study, GFAP was used as a tool to assess astrogliosis during seizures. Rats injected with Li/PIL showed a massive expression of GFAP in the CA1, CA3, and hilar regions of the hippocampi, which were shown to be the same sites of injury by histopathological examination. Our findings harmonize with previous studies that revealed a rapid amplified GFAP expression in various animal models of epilepsy, including PTZ (Torre et al., 1993; Sedky et al., 2017), electrical kindling (Stringer, 1996; Miyazaki et al., 2003), and kainic acid (Bendotti et al., 2000; Choi et al., 2003; Glushakova et al., 2012; Lee et al., 2012). Similarly, Alese and Mabandla (2019) recorded a marked increase in GFAP hippocampal expression 30 min following PIL administration in rats with or without a history of prolonged febrile seizure. Genetically induced extensive astrogliosis in mice disrupted glutamate uptake and triggered spontaneous seizures (Robel et al., 2015). In the same context, a previous study synchronizes with our finding, where the anticonvulsant activity of TMZ in PTZ-treated mice was associated with a marked decrease in hippocampal GFAP expression (Sedky et al., 2017).

In the current study, enhanced GFAP expression was also accompanied with an increase in the hippocampal glutamate level as well as a marked reduction in mRNA expression of both GLT-1 and GLAST. In agreement with these results, astrocytes activation was coupled with decreased GLT-1 and extracellular glutamate flooding in models of peripheral nerve injury (Cavaliere et al., 2007; Cirillo et al., 2011). Furthermore, the expression of GLAST and GLT-1 has been diminished in the cortices as well as in the hippocampi of rats with genetic absence seizures (Dutuit et al., 2002) and in a mouse model of focal epilepsy (Ingram et al., 2001).

Interestingly, GFAP and *p*-ERK1/2 were found to be co-expressed in the hippocampi of mice subjected to PIL-induced status epilepticus after 6 h. However, GFAP, but not *p*-ERK1/2, was still expressed after 3 days (Li et al., 2009). Similarly, Choi et al. (2003) reported a rapid and transient *p*-ERK1/2 increase in hippocampal neurons and astrocytes following kainic acid–induced seizures. These results go in line with the present study and suggest the possible contribution of *p*-ERK1/2 in the protective effect of astrogliosis in the early stages of epilepsy. In the same context, *p*-ERK1/2 was previously claimed to mediate GLT1 expression in astrocytes (Frizzo et al., 2007); however, its elevated level attained after Li/PIL injection in the present study was probably not enough to increase the gene expression of glutamate transporters. In addition, during astrogliosis, the activated protease, calpain-I, was previously accused of astrocyte transporters degradation (Cavaliere et al., 2007). Coherent with these results, Berkeley et al. (2002) demonstrated that PIL caused initial ERK activation, while its inhibition had not prevented PIL-induced seizures, yet increased its severity. The further increase in *p*-ERK1/2 observed after TMZ may be a partial mechanism through which it could increase mRNA expression of astroglial transporters and, consequently, glutamate uptake.

Simultaneously, mitochondrial dysfunction associated with the obvious alterations in mitochondrial oxidative status exerts an important role in modulating glia–neuron interplay (Jain et al., 1991; Wallace et al., 1992; Lin et al., 2020). Li/PIL injection in the current study provoked a state of mitochondrial oxidative burst as manifested by diminished TAC and GSH along with enhanced lipid peroxidation. In the same context, Waldbaum et al. (2010) reported a persistent perturbation of mitochondrial redox status during acute and chronic phases of Li/PIL-induced epilepsy. Under the condition of ATP deficiency coupled with mitochondrial oxidative damage as demonstrated herein, the expression and function of astroglial transporters are disrupted (Frizzo et al., 2007). Though extracellular ATP release from damaged neurons succeeded in inciting GLT1 expression *via* ERK1/2 activation (Neary et al., 1999, Neary et al., 2003), the reduced ionic gradient following energy failure may refute the driving force essential for glutamate uptake (Kauppinen and Swanson, 2007). Accordingly, synaptic glutamate increase was ascribed to glutamate uptake impairment (Colangelo et al., 2014) paralleled with glutamate efflux by the reversed action of transporters (Seki et al., 1999; Phillis et al., 2000). Additionally, the enhancement of hippocampal large-conductance Ca^2+^-activated K^+^ channels in response to redox status change may pose a plausible mechanism toward increasing hippocampal neuronal excitability after exposure to extracellular glutamate (Waldbaum and Patel, 2010).

TMZ was effective in restoring mitochondrial oxidative homeostasis as evident by the enhanced TAC and restored GSH. Ultimately, it cured lipid peroxidation, an effect that reflected on mitochondrial efficacy in ATP production and, consequentially, function and expression of astroglial glutamate transporters. The activity of TMZ on glial uptake of glutamate was previously documented in an *in vitro* study using rat retinal Müller cell line. It revered glial transporter inhibition and protected the retina from ischemia-induced excitotoxicity (Payet et al., 2004). In agreement with our results, Jain et al. (2011) attributed the anticonvulsant activity of TMZ following PTZ-induced kindling in mice mainly to its robust antioxidant activity. The well-recognized antioxidant properties of TMZ are thought to be mediated indirectly by boosting the anti-oxidant enzymes (Tikhaze et al., 2000). This effect may be linked to the ability of TMZ to increase *p*-AMPK, which largely participates in restoring neuronal energy balance (Ronnett et al., 2009). AMPK activators were reported to induce nuclear retention of antioxidant transcription factors as forkhead box O1 (Yun et al., 2014) and nuclear factor erythroid 2–related factor 2 (Joo et al., 2016). In the same context, TMZ increased the ATP/ADP ratio in the hippocampus of diabetic epileptic rats (Mohamed et al., 2020). In addition, it restored ATP synthesis after cerebral mitochondrial respiration inhibition by cyclosporin (Zini et al., 1996).

Intracellular Ca^2+^ accumulation following excitotoxic insults was reported not only to participate in further mitochondrial ROS production and ATP synthesis inhibition but also inevitability to the activation of membrane transition pores and Cyt c release, leading to apoptotic neuronal death (Sullivan et al., 2005), as demonstrated herein by the increase in caspase-3 activity. The increase in mitochondrial UCP2 observed herein and previously (Diano et al., 2003) is postulated to reduce ROS production and guard against neuronal death. Usually, enhanced UCP2 in response to various neuronal stress is one of the neuroprotective responses, by increasing the number of mitochondria and consequently ATP production. However, these beneficial effects were obviously established after 5 days in a Li/PIL animal model (Dutra et al., 2018). Furthermore, hippocampal neurons employ another survival response *via* boosting ERK expression (Berkeley et al., 2002) to act in a mutual intervention with ERK induced in astrocytes during initial astrogliosis. The implication of ERK1/2 in neuroprotection in response to excitotoxicity was previously attributed to its ability to upregulate the antiapoptotic Bcl2 (Ortuño-Sahagún et al., 2014). In addition, phosphorylation of Kv4.2, fast-inactivating A-type potassium channels present postsynaptically, by activated ERK1/2 enables their localization to areas of hyperactivity in the hippocampus. Consequently, they accelerate repolarization and limit firing of action potential to guard against lethal seizures and neuronal death (Berkeley et al., 2002).

In the present study, the success of TMZ in limiting seizure incidence was associated with a marked reduction in apoptotic neuronal death rate as manifested by the obvious decrease in hippocampal Cyt c and caspase-3 activity. The anti-seizure as well as the antiapoptotic effects of TMZ could be linked to the potentiating effect of ERK phosphorylation in both neurons and glial cells. In addition, the effect of TMZ on AMPK could participate in its antiapoptotic activity *via* endorsing high mitochondrial membrane potential to preserve Ca^+2^ homeostasis (Zhang et al., 2017). In agreement with these results, Liu et al. (2016) reported that TMZ could protect cardiomyocytes against ischemic injury *via* activation of both AMPK and ERK. Intriguingly, purified rat brain mitochondria have been shown to express binding sites for TMZ (Morin et al., 2000). Former *in vitro* studies advocated that TMZ could inhibit mitochondrial permeability transition pores' opening (Rothstein et al., 1992; Argaud et al., 2005) and thus hinder apoptotic neuronal loss following hyperexcitability.

In conclusion, TMZ showed a promising anti-seizure potential that was evident on seizure score as well as on various biochemical indicators. The beneficial actions of TMZ could be attributed to its ability to decrease mitochondrial oxidative damage as well as glutamate accumulation, parallel to a positive modulation of *p*-ERK1/2/p-AMPK signaling. Also, it succeeded in reducing ATP-dependent energy disruptions as well as astrocytes activation. This was all reflected as a reduction in neuronal apoptosis and preserved cellular integrity.

## Data Availability

The original contributions presented in the study are included in the article/Supplementary Material, further inquiries can be directed to the corresponding author.
