# Supplementary figures and images for: Trimetazidine Modulates Mitochondrial Redox Status and Disrupted Glutamate Homeostasis in a Rat Model of Epilepsy
Source: Front Pharmacol. 2021 Oct 8;12:735165. doi: 10.3389/fphar.2021.735165 (PMC8531497; doi:10.3389/fphar.2021.735165)

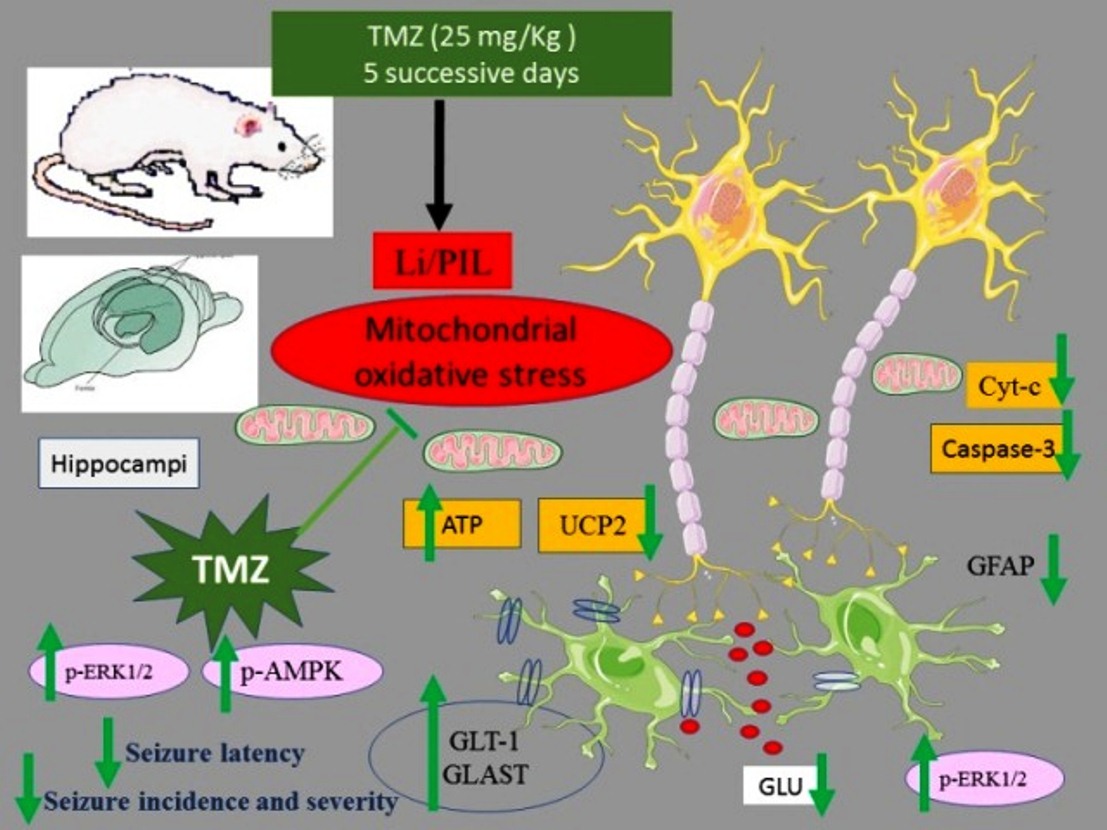

Supplement: Supplementary file 1 [file Image1.JPEG]
